# Advanced Development of Supercritical Fluid Chromatography in Herbal Medicine Analysis

**DOI:** 10.3390/molecules27134159

**Published:** 2022-06-29

**Authors:** Min Chen, Shan-Shan Wen, Rui Wang, Qing-Xuan Ren, Chen-Wan Guo, Ping Li, Wen Gao

**Affiliations:** State Key Laboratory of Natural Medicines, China Pharmaceutical University, No. 24 Tongjia Lane, Nanjing 210009, China; chenmin1551@163.com (M.C.); wenss0320@163.com (S.-S.W.); w.rx60@163.com (R.W.); xuan533129@163.com (Q.-X.R.); gcw0714@163.com (C.-W.G.)

**Keywords:** supercritical fluid chromatography, herbal medicines, stationary phase, mobile phase, multidimensional chromatography, application

## Abstract

The greatest challenge in the analysis of herbal components lies in their variety and complexity. Therefore, efficient analytical tools for the separation and qualitative and quantitative analysis of multi-components are essential. In recent years, various emerging analytical techniques have offered significant support for complicated component analysis, with breakthroughs in selectivity, sensitivity, and rapid analysis. Among these techniques, supercritical fluid chromatography (SFC) has attracted much attention because of its high column efficiency and environmental protection. SFC can be used to analyze a wide range of compounds, including non-polar and polar compounds, making it a prominent analytical platform. The applicability of SFC for the separation and determination of natural products in herbal medicines is overviewed in this article. The range of applications was expanded through the selection and optimization of stationary phases and mobile phases. We also focus on the two-dimensional SFC analysis. This paper provides new insight into SFC method development for herbal medicine analysis.

## 1. Introduction

Herbal medicines (HMs), the main carrier of traditional Chinese medicines (TCMs), have been widely used for disease treatment and human health care [1]. Nowadays, some effective natural components such as berberine, ephedrine, and artemisinin have been developed into modern medicines. However, most HMs are used in multiple component forms, such as *Ginkgo biloba* extracts, ginseng preparations, and *Ganoderma lucidum* spore powder [2]. Therefore, an analysis of the multiple chemical constituents in HMs not only provides opportunities for new drug discovery but is also key to the quality control of HMs.

Liquid chromatography–mass spectrometry (LC-MS) as well as gas chromatography–mass spectrometry (GC-MS) have been widely used for complicated component analysis [3]. Although various MS detectors provide high sensitivity and resolution for the identification, quantification, and confirmation of analytes, the main drawback of these detectors is the matrix effect, which can be solved with a previous chromatographic separation [4]. As HMs usually comprise hundreds of constituents that belong to diverse chemical and physical properties, the choice of chromatographic type depends to a large extent on the properties of the analyte (polarity, volatility, etc.). LC is the most popular separation strategy for TCM research [5], possibly because of its various separation mechanisms, such as reversed-phase, normal-phase, hydrophilic interaction chromatography, ion exchange, and others. Meanwhile, the use of sub-2 μm particle-size columns significantly increases separation efficiency [6], and comprehensive two-dimensional LC improves peak capacity [7], making LC suitable for the qualitative and quantitative analysis of multiple components in HMs [8].

Supercritical fluid chromatography (SFC) is a chromatographic technique that uses a supercritical fluid, a low-viscosity solvent, as the mobile phase. The most commonly used supercritical fluid is carbon dioxide (scCO_2_), which has a similar polarity to hexane or pentane [9]. Klesper [10] first used supercritical fluids in chromatography in 1962, and capillary column SFC (cSFC) was developed about 20 years later [11,12,13]. Considered an advanced application of gas chromatography (GC), cSFC is typically combined with the flame ionization detector (FID) and requires pure supercritical fluid as the mobile phase. This property limits it to only hydrophobic compounds, and it has a narrow scope of application, which is possibly the reason that cSFC disappeared in the 1990s [14]. After the development of SFC equipment that overcame the deficiencies in instrumental stability and detection sensitivity, modern SFC was resurgent. To date, the commercial SFC instrumentation, such as the Waters ACQUITY UPC^2^, the Agilent 1260 Infinity Hybrid UHPLC/SFC, the Shimadzu online SFE-SFC system, or the Jasco SFC Hybrid system between analytical and preparative SFC [15], improves a new chemical separation strategy in HM analysis.

Compared with HPLC, due to its higher flow rate and lower viscosity, SFC has the following advantages: (a) a lower pressure at the high flow rate, (b) a shorter analysis time for high-throughput analysis, (c) a good separation efficiency and unique selectivity, and (d) less organic solvent consumption for environmental friendliness. However, it has some limitations: (a) more operating parameters (the flow rate, column temperature, and pressure were coupled such that one of them changed as the others changed) and (b) a strong solvent effect (the selection of dissolution solvents for polar compounds is limited) [16]. Compared with GC, SFC enables a wider selection range of operating conditions and efficient separation of thermally labile compounds. Furthermore, SFC required fewer organic solvents, and the peak broadening of SFC is narrower than that of LC. For example, for the analysis of the indole and oxindole alkaloids in *Mitragyna speciosa* plants, the established UHPLC method required acetonitrile and water with ammonium acetate, which resolved the major alkaloids in 30 min but was not specific to the mitragynine diastereoisomers. The alkaloid diastereoisomers without derivatization could not be separated by the established GC method (18 min), and the required high temperature for alkaloids analysis in GC imposed a severe restriction on the adjustment of some parameters for resolution. The eight major compounds, including two pairs of diastereoisomers, were successfully separated by SFC in 8 min, which is faster and more efficient than HPLC and GC when using the UV detector [17].

The SFC separation of multi-components containing various types or classes in HMs mainly depends on the selection of stationary phases and the optimization of mobile phases. Meanwhile, its analytical capacity could be improved based on the two-dimensional mode. Therefore, we focus on the selection of the above-mentioned factors and summarize the application of SFC in HM analysis (2010–2021).

## 2. Selection of Stationary Phases

West and Lesellier [18,19,20] have published a series of articles to study SFC stationary phases. The linear solvation energy relationship (LSER) model illustrates column properties using Abraham descriptors, as described by the Equation (1):

log *k* = *c* + *e*E + *s*S + *a*A + *b*B + *v*V (1)

The capital letters indicate interactions between solutes and columns. The E, S, A, B, and V represent the charge transfer interaction, dipole–dipole interaction, hydrogen-bond donor, hydrogen-bond acceptor, and dispersion. The lower-case letters represent the coefficient values, and *c* is the intercept term of the model.

This model is specific to neutral analytes. Therefore, the model could be upgraded and two descriptors are introduced for ionic compounds [21], as follows Equation (2):

log *k* = *c* + *e*E + *s*S + *a*A + *b*B + *v*V + *d*^−^D^−^ + *d*^+^D^+^ (2)

D^−^ represents the ionic interaction performed by anionic and zwitterionic ions, and D^+^ represents the ionic interaction generated by cationic and zwitterionic ions. For chiral stationary phases [22,23], two additional descriptors, the flexibility of the analytes (F) and globularity (G), were introduced to provide complementary information, as follows Equation (3):

log *k* = *c* + *e*E + *s*S + *a*A + *b*B + *v*V + *f*F + *g*G (3)

A positive coefficient shows the interaction between the solute and the stationary phase, while a negative coefficient represents the interaction with the mobile phase. The coefficient is numerically larger, indicating a stronger interaction. This theory provides guides for selecting appropriate chromatographic columns.

### 2.1. Non-Polar Stationary Phases

The non-polar stationary phases, such as the C8, C18, and C30 columns, consist of alkyl-bonded stationary phases that do not have hydrophilic groups. These columns are suitable for the separation of hydrophobic compounds such as lipids, carotenoids, terpenes, and many substances with low polarities (*e* and *v* are positive). Polar analytes generally show poor retention behaviors (*s*, *a*, and *b* are negative) and have poor peak shapes [19,24].

SFC was sometimes considered reverse-phase liquid chromatography (RPLC) in this condition [15], but it is worth noting that the mobile phase of SFC is usually a mixture of low-polar CO_2_ and a more polar modifier. During the gradient elution procedure, the proportion of modifiers is gradually increased. Therefore, the polarity of the mobile phase changes from low to high, and it is not similar to RPLC.

Carotenoids are the natural pigments with health benefits in plant seeds. The C18 and C30 columns are the most commonly used stationary phases for the separation of carotenoids. The Giuffrida group [25] performed an SFC-APCI-QQQ-MS method for the determination of apocarotenoids in *Capsicum chinense*. In this study, 25 apocarotenoids were identified on a novel C30 fused-core column with sub-2 μm particles within 5 min, including 14 free apocarotenoids and 11 apocarotenoids fatty acid esters. Furthermore, an online supercritical fluid extraction–supercritical fluid chromatography–mass spectrometry (SFE-SFC-MS) system was then developed for the extraction and identification of carotenoids in *Capsicum chinense* [26]. The extraction process had no saponification step and was optimized by changing the pressure, temperature, and modifier percentage. The conditions for the complete extraction of all carotenoids were 150 bar, 80 °C and 20% MeOH (extraction yield about 50%). Twenty-one carotenoids were extracted and identified on the novel C30 fused-core column within 17 min, including free, monoester, and diester carotenoids. The methodology was also applied to the characterization of carotenoids and apocarotenoids in *Solanum betaceum* fruits [27]. Compared to the traditional YMC C30 column, the novel C30 fused-core column could provide a shorter elution time of about 6 min and a better separation of carotenoid diesters. In brief, SFC is suitable for the carotenoid separations due to the short analysis time, efficient resolution, and low organic solvent consumption.

The orthogonality between SFC and RPLC was investigated by comparing the elution order when identifying sesquiterpenes and other components from *Matricaria chamomilla* and *Chamaemelum nobile* extracts. The elution orders of each peak in SFC and RPLC are inverse, demonstrating the high orthogonality of the two chromatographic techniques [28].

### 2.2. Polar Stationary Phases

SFC is generally performed as a normal-phase liquid chromatography (NPLC) mode using a polar stationary phase. The polar stationary phases include bare silica gel, 3-aminopropyl bonded silica (NH_2_), 3-cyanopropyl bonded silica (CN), propanediol bonded silica (Diol), and others. The moderately polar stationary phases include numerous aromatic stationary phases and short-chain alkyl stationary phases, such as phenylhexyl (C6PHE), phenylpropyl (C3PHE), pentafluorophenyl (PFP), and diphenyl (DP) bonded silica. These columns offer more options for SFC separation. These stationary phases are suitable for the analysis of polar compounds such as saponins and phenolics (*a*, *b*, *e*, *s*, and *d*^+^ are positive, while *v* and *d*^−^ are negative) [20]. If the stationary phase contains silanol groups, the alcohol modifier converts the silanol to silyl ether, altering the chromatographic retention and selectivity. This phenomenon also occurs in hydrophilic interaction liquid chromatography (HILIC) or RPLC. However, the mobile phase with significant amounts of water quickly removes the silyl ethers. Therefore, it is often recommended to store SFC columns in pure carbon dioxide to prevent changes in the stationary phase properties [29].

The specific SFC stationary phase, the 2-ethylpyridine (2-EP) column, was designed for the analysis of basic compounds such as alkaloids without the use of basic mobile phase additives. The nitrogen atoms of the pyridine moiety of the stationary phase possess hydrogen bonding acceptor capabilities. Under the acidic conditions generated by methanol and scCO_2_, the 2-EP moiety becomes protonated and positively charged, creating electrostatic repulsion with the analytes and forming π-π interactions with the basic analytes. The interactions mentioned above affect the retention behavior of the alkaloids. The hydrogen bonding interaction of the stationary phase is strongly influenced by the modifier. Therefore, using a modifier with hydrogen bond donor properties, such as methanol, weakens the hydrogen bonding interaction between the stationary phase and the analytes. In contrast, the use of hydrogen bond acceptor modifiers, such as acetonitrile, resulted in excessive retention [30,31].

Saponins are an important component of the active ingredients in HMs. Huang et al. [32] reported the isolation of triterpenoid standards (kudinosides, stauntosides, and ginsenosides) and triterpenoid extracts from *Ilex latifolia* leaves, *Panax quinquefolius* roots, and *P. ginseng* roots. The polar characteristics of triterpenoids resulted in no retention on the SB-C18 column, while the ZORBAX RX-SIL column achieved the best triterpenoid separation performance by using CO_2_, MeOH, H_2_O, and 0.05% (*v*/*v*) formic acid as the mobile phase. The SFC method was faster than the HPLC method, and the elution order in the SFC method was opposite to that in the HPLC method. The saponins with fewer sugar groups were eluted first, while saponins with more sugar groups were strongly retained. The results indicated the complementarity of the two separation techniques.

The methoxylation or ethoxylation of the hydroxyl group at the C-22 position of furostanol saponins is usually observed when it reacts with lower alcohols under appropriate conditions. Yang et al. [33] analyzed the furostanol saponins in the *Dioscorea zingiberensis* rhizome based on the Diol column using methanol containing 0.2% NH_4_OH and 3% H_2_O as the modifier, which minimizes the degree of derivatization. Furthermore, furostanol saponins were well-identified by SFC based on the number and type of sugars. The polarity of glucosyl was stronger than that of rhamnosyl, and the polarity of furostanol saponins became stronger as the number of sugar groups increased. Therefore, the retention time of saponins with high polarities became longer. However, the isomers could not be separated.

**Figure 1:**
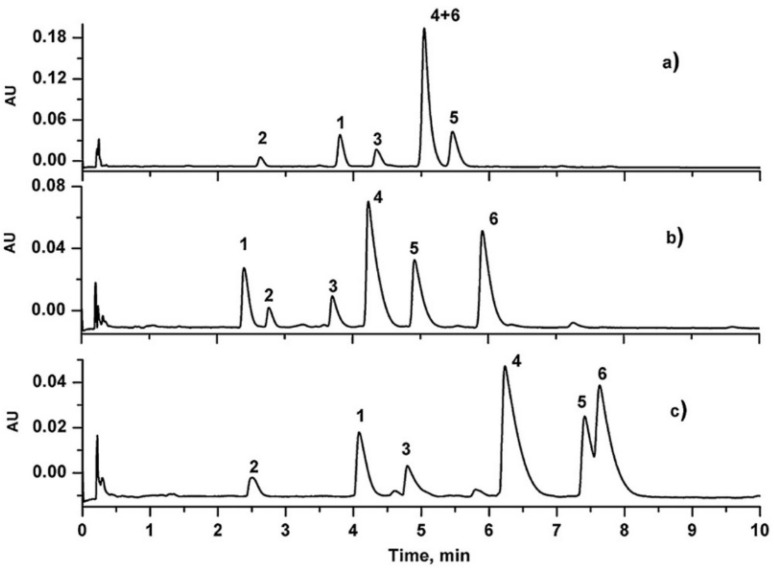
Optimization of stationary phases for six standard alkaloids separation. (**a**) BEH, (**b**) BEH 2-EP, and (**c**) CSH PFP columns. Analytes: (1) compound 1, (2) euonymine, (3) wilfornine D, (4) wilforgine, (5) wilforine, and (6) hyponine E. Adapted from [31] with permission. Copyright 2015, Elsevier.

Seventy-one sesquiterpene pyridine alkaloids in *Tripterygium wilfordii* root bark extract were successfully analyzed on the ACQUITY UPC^2^ BEH 2-EP column in combination with an MeOH modifier without additives in less than 10 min. Alkaloids were strongly retained on the BEH column due to the ion-exchange interactions between alkaloids and the silanol groups on the surface of the stationary phase. Broader peaks were observed on the CSH PFP (charged surface hybrid silica bonded with a fluoro phenyl group) column [31] (Figure 1).

Polar stationary phases have also been used for the separation of hydrophobic compounds. Hou et al. [34] used the Torus 2-PIC column for the separation of lipids in *Coix lacryma-jobi* ripe caryopsis with different geographical origins. The HSS C18 SB column had strong retention, the CSH FP column had coelution, and the other polar stationary phases had poor separation. The same column was also used to explore the lipidomic differences of three Panax species (*P. ginseng*, *P. quinquefolius*, and *P. notoginseng*) [35].

### 2.3. Chiral Stationary Phases

Chiral separation is mainly based on the formation of a transitional diastereomeric complex between the analytes (SAs) and chiral selectors (SOs) on the chiral stationary phases (CSPs), relying on modifiers and additives for the separation. CSPs involve at least three different combinations of physiochemical properties, including hydrogen-bonding interactions, dipole–dipole interactions, π-π interactions, electrostatic interactions, hydrophobic interactions, and spatial interactions [36,37].

The chiral stationary phase, designated a UHPC-(*S*, *S*)-Whelk-O1 column, was used to separate a *R*- and *S*-goitrin mixture in *Isatis indigotica* root, *Baphicacanthus cusia* root, and Ban Lan Gen powder formulations within 6 min. This column accomplished this with a suitable resolution and an almost eight-fold increase in speed compared to the NPLC method [38]. Phytocannabinoids are derived from the *Cannabis sativa* L. species. Most of them are chiral and exist in the single-enantiomeric format. The Gasparrini group [39] utilized a UHPC-(*S*, *S*)-Whelk-O1 column and a UHPC-(*R*, *R*)-Whelk-O1 column for the enantio- and chemo-selective separation of phytocannabinoids by UHPSFC. The method was based on the “Inverted Chirality Columns Approach” (ICCA) according to the reciprocal principle [40]. The elution order of the enantiomers was reversed by switching two chiral columns with the same SO and opposite configuration. This method shows great potential for the identification of enantiomers without standards.

### 2.4. Other Stationary Phases

With the development of technology and the increase in experimental demand, several novel stationary phases are being developed [41,42,43,44,45,46,47,48]. The Chou group [41] covalently bonded 1-octyl-3-propylimidazolium chloride on silica gel to produce an ionic liquid-functionalized stationary phase. Compared with the C18 stationary phase, the column has a longer chain length of the alkyl group to increase hydrophobic interaction and can separate acidic, basic, and neutral compounds simultaneously. Complete separation occurred when CO_2_ and MeOH were used as the mobile phase. Electrostatic and hydrogen-bonding interactions are essential for the separation. This implies that the addition of water and trifluoroacetic acid increases the elution strength of the mobile phase. Neutral compounds with weak hydrophobic interactions are eluted first, while acidic and basic compounds are more strongly retained. Due to the complexity of the multi-components, there is an urgent need for novel stationary phases for HM analysis that can separate complex components simultaneously.

## 3. Selection of Mobile Phases

### 3.1. Modifiers

Due to the low polarity of scCO_2_, the variety of compounds analyzed with SFC is limited. Modifiers are added to adjust the solvent strength of the mobile phase. The high miscibility of CO_2_ with many organic solvents contributes to the expansion of the application. Short-chain alcohols are commonly used as modifiers in SFC, such as methanol, ethanol, and isopropanol, among which methanol is the most commonly used. It is important to note that when used as a modifier methanol can contain up to 10% water, while isopropanol can contain up to 50% water [49].

Modifiers affect chromatographic retention in several ways: (a) improving mobile phase polarity and improving mobile phase eluting power, (b) changing mobile phase density, (c) modifiers adsorb to the surface of the stationary phase, thus changing the properties of the stationary phase, which many articles have investigated [50,51,52,53], and (d) masking the active site on the stationary phase. Free silanols on the stationary phase surface have both hydrogen-bonding acceptor and hydrogen-bonding donor capabilities that can affect the analyte peak shape. Alcohols also have both hydrogen-bonding acceptor and donor properties, so they can minimize this effect. Acetonitrile has a weak ability to cover silanol groups, so it can be mixed with methanol as a modifier to improve the separation ability [16,54].

Liu et al. [55] used SFE-SFC-MS/MS for the analysis of phenolic compounds. Three modifiers, methanol, acetonitrile, and a mixture of methanol and acetonitrile (2:1, *v*/*v*), were investigated. The polar protic solvents are more conducive to the formation of hydrogen bonds, and the charge separation in the ESI droplet is more stable for the separation of polar phenolic compounds. The significantly increased responses of the majority of the target compounds and the separation efficiency followed the order methanol, a mixture of methanol and acetonitrile (2:1, *v*/*v*), and finally acetonitrile. Therefore, methanol was identified as a mobile phase modifier for the separation of phenolic compounds.

### 3.2. Additives

Unlike modifiers, additives are added to the mobile phase to improve chromatographic performance by competing with solutes for adsorption sites on the surface of the stationary phase. In general, acidic additives (formic, acetic, trifluoroacetic, and phosphoric acid, etc.) can be selected for the analysis of acidic compounds, while basic additives (isopropylamine, diethylamine, ammonium hydroxide, etc.) are selected for the analysis of basic compounds. Salt additives such as ammonium formate and ammonium acetate can be applied to amphoteric compounds. When using different additives and modifiers, we should pay attention to increasing the column equilibration time [56,57]. The addition of water separates the more polar compounds. Ashraf-Khorassani et al. [58] proposed that the water additive altered the properties of the bare silica columns, thus generating an HILIC-like retention mechanism. The analytes are partitioned between the water in the mobile phase and the water adsorbed on the surface of the stationary phase.

For bare silica columns, an additive such as ammonium hydroxide is added to the modifier, which acts as a competitor for the active site on the stationary phase surface and masks the residual silanol group on the stationary phase. In this case, the main interaction is hydrogen bonding between the hydrophilic compounds and the methanol or the basic additive adsorbed on the stationary phase surface. At this point, the hydrogen bonding interactions have a large impact on chromatographic retention. If the analyte has a large number of hydrogen bonding donor or acceptor groups, it is difficult to elute from the stationary phase [59]. A recent study reported that the presence of both water and ammonium hydroxide in the methanol modifier, in an in situ formation of HCO_3_^−^ produced through the chaotropic effect, improves the separation of hydrophilic compounds and provides excellent chromatographic performance [60].

Phenolics are an important class of HM constituents, including phenolic acid, flavonoids, isoflavones, lignans, etc. Phenolics contain one or more phenolic hydroxyl groups and are acidic. SFC has been used successfully for the separation of phenolics in various matrices. The C18 column is widely used for separating phenolics in RPLC with remarkable performance. However, it is not suitable for the SFC separation of phenolics directly. For the acidic properties of phenolics, serious peaking tailing could be observed. The selection of a suitable additive, which increases the acidity of the modifier, plays a significant role in SFC method development.

**Figure 2:**
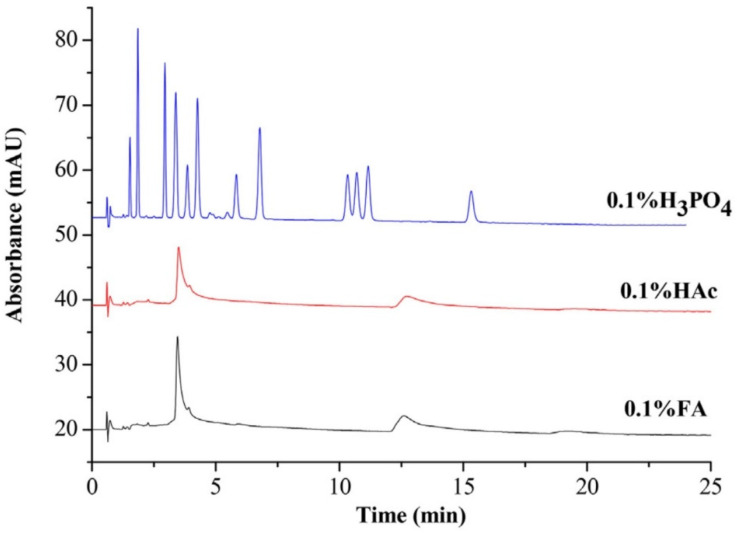
Effect of the addition of an acidic additive to the mobile phase on the separation of flavonoids. Column: ZORBAX RX-SIL column (150 mm × 4.6 mm, 5 μm); mobile phase: supercritical carbon dioxide (scCO_2_) and MeOH containing 0.1% formic acid (FA), 0.1% acetic acid (HAc), or 0.1% phosphoric acid (H_3_PO_4_). Adapted from [61] with permission. Copyright 2017, Elsevier.

Flavonoid aglycones and their glycosides are an interesting class of both hydrophobic and hydrophilic compounds in phenolics. The peak shape could be improved by the addition of acidic additives. Formic, acetic, and phosphoric acid were investigated for flavonoid analysis on the ZORBAX RX-SIL column (Figure 2). When formic and acetic acid were used as additives, the flavonoids could not be eluted because the flavonoids interacted strongly with the stationary phase. Phosphoric acid could compete with flavonoids for the active sites, facilitating the elution of flavonoids. Compared with the HPLC method, the SFC method can provide separation about three times faster [61]. Phosphoric acid is also applied for isoflavone separation in SFC. The BEH column with 0.05% phosphoric acid as an additive provided better peak separation and less baseline drift. Aglycones eluted earlier than the glycosides. The developed method was applied to the analysis of dietary supplements containing *Glycine max* bean, *Trifolium pratense* blossom, and *Pueraria lobata* root for 8 min [62]. Sun and co-workers [63] used oxalic acid as an additive in the modifier (with MeOH/ACN, 50/50, *v*/*v*) in UHPSFC-QTOF/MS for the efficient separation of 51 prenyl flavonoids, including aglycone and glycosides from *Epimedium* species for the first time, then the developed method was successfully applied for the differentiation and quality assessment of *Epimedium* species. Gao et al. [64] optimized an ionic liquid (IL) called 1-butyl-3-methylimidazolium tetrafluoroborate ([bmim][BF_4_]) as an unconventional additive for the separation of six structurally similar flavonoid aglycones. The addition of IL improved the resolution and increased the retention factors. The authors proposed that a new hydrogen-bonding interaction was formed between flavonoids (with hydrogen-bonding acidity) and ionic liquid (with hydrogen-bonding basicity) to enhance the separation. Meanwhile, 0.1% methanesulfonic acid (MSA) in methanol was used for the separation of nine flavonoid standards, including aglycones and glycosides on the polar stationary phase Torus DEA column, then the aglycones were eluted first, followed by their glycoside forms, and glycosides with smaller sugar groups were eluted more easily [65].

Alkaloids are a class of basic organic compounds containing nitrogen atoms that exist in nature and have significant biological activity. SFC analyses of alkaloids and other basic compounds produce peak shape distortions such as trailing, fronting, and splitting, resulting in poor chromatographic performance. The reasons for this situation are: (a) The most widely used modifier for SFC is methanol. The mobile phase is acidic (apparent pH is about 4–5) due to the reaction of methanol and scCO_2_ to form methyl carbonate. Under such acidic conditions, alkaloids can form alkaloid cations in the mobile phase, which can interact with the negatively charged silanol groups remaining on the surface of the polar stationary phase through ion exchange. Therefore, a strong retention is produced on the column. (b) scCO_2_ reacts with the amino groups in the basic compound to form carbamic acid, a reaction that strongly depends on the spatial site resistance of the amino substituent. In the presence of methanol, the conversion to methyl carbonate preferably occurs so that the conversion of carbamic acid is usually not observed [30,31].

To solve the above-mentioned problem of the chromatographic separation of alkaloids, basic additives can be added to the mobile phase, which compete for the active sites on the stationary phase surface, mask the silanol groups, and thus improve the chromatographic separation.

Yang et al. [66] utilized the 1-AA and Diol columns for the separation of rhynchophylline and isorhynchophylline, and corynoxine and corynoxine B, present as two pairs of 7-epimeric spiro oxindole alkaloids (SOAs) in *Uncaria macrophylla*. The 7-epimeric SOAs trended to isomerize in the protic MeOH compared with the aprotic ACN. Therefore, ACN is a significant solvent as the modifier for the SFC separation. It was found that 0.1% diethylamine as the additive on the 1-AA column and 0.1% ammonium hydroxide on the Diol column are suitable for UV and MS detection, respectively. The developed method facilitated the quality control of *Uncaria macrophylla*. Huang et al. [67] found that 0.2% (*v*/*v*) NH_4_OH was the optimal additive compared to diethylamine and trimethylamine for the separation of alkaloids on the PFP column in *Mahonia bealei* stem, root, leaf, and seed extracts. Indeed, water could improve the peak shape and elution in this study. The SFC method could provide separation about 13 times faster than the LC method, showing that SFC could be an alternative separation method.

Jiang et al. [68] used deep eutectic solvents (DESs) consisting of choline chloride (hydrogen bond acceptor) and glycerol (hydrogen bond donor) for the separation of 10 isoquinoline alkaloids. DES as a silanol blocker to occupy the residual silanol group on the surface of the stationary phase competes with the isoquinoline alkaloids for the active sites and could be used to improve chromatographic separation and prevent serious peak tailing for the analysis of isoquinoline alkaloids. It should be noted that the effect of DES on the analysis of isoquinoline alkaloids was mainly attributed to the hydrogen bond acceptors. The established method was then applied for the analysis of the alkaloids in the *Sinomenium acutum* stem rattan, *Corydalis yanhusuo* rhizome, *Coptis chinensis* rhizome, *C. deltoidei* rhizome, *C. teeta* rhizome, *Mahonia bealei* stem, *M. fortune* stem, *Phellodendron chznense* bark, and *Stephania tetrandra* root extracts.

**Figure 3:**
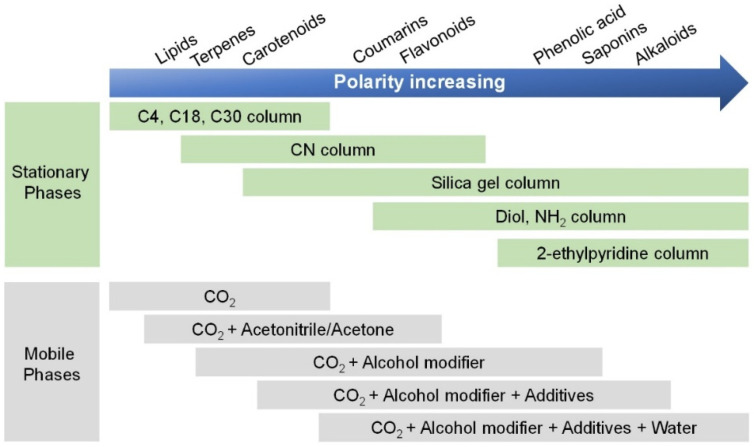
Overview of the specific stationary phases and mobile phases that match the solute. As the polarity of the solute increases, so does the polarity of the stationary phase and mobile phase.

To achieve the best separation of components with different polarities in the sample in the shortest time, gradient elution is usually preferred, which could increase the elution strength of the mobile phase by adjusting the proportion of the modifiers in the mobile phase. Gradient elution can make the components with different properties in a complex sample separate well according to their appropriate capacity factor, *k*, which can shorten the analysis period, improve the separation ability, increase the sensitivity, and improve the peak pattern. Taguchi and co-workers [69] developed a unified chromatography to successfully analyzed water- and fat-soluble vitamins using low to 100% ratios of modifiers. Fat-soluble vitamins were well-retained on the C18 SB column for the hydrophobic interaction. Meanwhile, the residual silanol groups on the stationary phase were successfully employed for the retention of hydrophilic compounds. This approach used methanol to separate 17 vitamins with diverse properties within 4 min, and the states, namely, supercritical, subcritical, and liquid, were continuously changed. Figure 3 demonstrates an overview of the current application areas of SFC, where the stationary phase and the mobile phase are selected according to the analytes. The SFC analysis (2010–2021) of various constituents from herbal medicine extracts is outlined in Table 1.

## 4. Two-Dimensional Systems of SFC

With the advantages of remarkable separation efficiency, high robustness, and wide compatibility, LC is currently a versatile technique. However, the ability of LC to handle complex samples is generally limited. Two-dimensional liquid chromatography (2D-LC) is considered an efficient alternative technique with a high peak capacity. It can be divided into two modes: offline and online modes. Analytes are collected and concentrated from the first dimension and then manually reinjected into the second dimension for further separation in offline mode. The online mode combines two separation techniques with special interfaces and is described as a comprehensive (noted LC × LC) and heart-cutting (noted LC–LC) mode [115].

Coupling LC and SFC provides various characteristics. The use of CO_2_ is much more convenient for fraction collection and can significantly reduce re-equilibration time in offline mode, regardless of SFC as the first- or second-dimension separation. Moreover, because of the ability to separate enantiomers, chiral SFC coupling with LC has a remarkable potential for the identification of both achiral and chiral components in complex samples. The properties of the CO_2_ expansion influence the analyte collection. Thus, the interfaces are necessary for the online SFC × LC mode. It is worth noting that strong solvent effects can easily cause peak broadening and deformation in the online LC × SFC mode, and there is still room for improvement. In addition, SFC × SFC is attractive because the use of CO_2_ in both separation modes could reduce mobile phase incompatibility and provide wider applicability to non-volatile and thermally labile compounds [116]. However, there are currently no commercially available instruments. Table 2 shows the two-dimensional SFC application of HMs.

### 4.1. SFC as the First-Dimension Separation

In general, NPLC × RPLC has high orthogonality. However, their mobile phases are incompatible. NPLC uses non-polar mobile phases, such as n-hexane, while RPLC uses polar mobile phases, including water and methanol. The different chemical properties of the mobile phases would cause peak distortion and splitting. SFC performs similar selectivity and reduces the incompatibility to NPLC. SFC × RPLC is considered an alternative technique to NPLC × RPLC.

Li et al. [117] used offline 2D SFC/RPLC to analyze amide alkaloids in *Piper longum*. Three SFC columns, named BEH, CSH FP, and XAmide columns, were used to develop a 2D SFC/SFC system. An HSS T3 column and three SFC columns were used to develop a 2D SFC/UHPLC system. Among these combinations, the orthogonality of the XAmide and HSS T3 column was up to 69.84%. It indicated that the 2D SFC/UHPLC system was the most suitable system for the separation of amide alkaloids. One to thirty-two fractions were collected from the SFC separation, dried and redissolved in H_2_O/ACN (3:7, *v*/*v*), then reinjected for UHPLC separation. Less than 50 peaks were separated by 1D UHPLC, while more than 340 peaks were separated by the 2D SFC/UHPLC system. The results illustrated the high orthogonality and peak capacity of the 2D SFC/UHPLC system.

In addition, Isabelle Francois [122] developed an online comprehensive SFC × RPLC method by connecting two separation modes through a two-position/ten-port switching valve equipped with two packed octadecyl silica (C18) loops. The addition of make-up water before the loops helped to aggregate analytes and reduce residual CO_2_ gas interference. Four 25 cm Princeton SFC cyanopropyl silica columns were used for the SFC separation because of the low viscosity of the supercritical fluids, and the 5 cm Zorbax SB C18 column was utilized for the separation of RPLC. The developed method was applied to the separation of psoralens and coumarins from lemon oil with high orthogonality.

### 4.2. SFC as the Second-Dimension Separation

Wei et al. [123] collected 40 fractions after the RPLC separation. The fractions were dried and concentrated with nitrogen, separated by the SFC separation using a C18 column, and then identified by mass spectrometry. Global natural product social molecular networking was used for data processing, which could significantly shorten the processing time. Finally, 229 bufadienolides and 2 new compounds were found in *Venenum Bufonis*. This method is particularly suitable for identifying structural isomers in complex samples.

Moreover, Gao et al. [125] constructed an online comprehensive NPLC × SFC platform that was connected by a 10-port, dual-position valve for the separation of the second metabolites in the fruiting bodies of *Ganoderma lucidum*. Most compounds were small and non-polar. Therefore, the CN and C18 columns were selected in NPLC and SFC separation mode, and 17 and 34 peaks were identified in 1D NPLC and 1D SFC separation, respectively. Coupling NPLC and SFC provides a high peak capacity and reduces incompatibility. A total of 250 peaks were observed, and the peak capacity increased to 350 in two-dimensional chromatography, while only 17 and 34 peaks were identified in one-dimensional NPLC and SFC separation, respectively. The system not only has good orthogonality but also has high throughput for the analysis of complex samples.

## 5. Conclusions

Chromatography is the mainstream technique for separating substances, and SFC provides many advantages over HPLC that cannot be substituted. SFC has become a significant supplement to HPLC due to its high efficiency, economy, and environmental friendliness. SFC-MS in particular is fast becoming a popular complement to LC-MS and GC-MS. Two-dimensional SFC has been reported successfully for the separation of complex analytes. The expansion of SFC applications cannot be achieved without the development of instruments, stationary phases, modifiers, and additives. This review mainly describes the application of SFC separations in herbal medicines. Depending on the properties of the analytes, suitable stationary phases, mobile phases, and detectors can be selected. Here, we should pay attention to the choice of volatile additives in SFC-MS. The concentration of additives in the modifier is generally 0.05–0.5%. In addition to conventional modifiers and additives, unconventional modifiers and additives can also be used to improve SFC separation and peak shape, making the peak shape sharper and more symmetrical, thereby expanding the application range of SFC. Further efforts are required for the development of new stationary phases and mobile phases, which will help SFC become a favorable analytical tool for a wider range of applications in the future.

## Figures and Tables

**Table 1:** Application of SFC in the analysis of natural products.

Categories	Analytes	Species (Part)	Stationary Phases (length × i.d., dp)	Modifier	Elution	Detector	Purpose	Ref.
**Lipids**	13 triacylglycerols	Soybean	three Chromolith Performance RP-18e columns (100 mm × 4.6 mm, –)	MeOH with 0.1% (*w*/*w*) ammonium formate	20 min; gradient elution	MS	Qualitative	[70]
	32 glycerides	*Coix lacryma-jobi* (ripe caryopsis)	Torus 2-PIC (100 mm × 3.0 mm, 1.7 μm)	MeOH:ACN (9:1)	8 min; gradient elution	MS	Qualitative	[34]
	24 lipids	*Panax ginseng*, *P. quinquefolius*, and *P. notoginseng*	Torus 2-PIC (100 mm × 3.0 mm, 1.7 μm)	MeOH	14 min; gradient elution	MS	Qualitative	[35]
**Carotenoids**	8 carotenoids	Microalgae and rosehip	Torus 1-AA (100 mm × 3 mm, 1.7 μm)	MeOH	7 min; gradient elution	DAD and MS	Qualitative Quantitative	[71]
	21 carotenoids	*Capsicum chinense*	Ascentis Express C_30_ (150 mm × 4.6 mm, 2.7 μm)	MeOH	16 min; gradient elution	MS	Qualitative Quantitative	[26]
	31 carotenoids	*Solanum betaceum* (fruit)	Ascentis Express C_30_ (150 mm × 4.6 mm, 2.7 μm)	MeOH	16 min; gradient elution	MS	Qualitative Quantitative	[27]
	25 apocarotenoids	*Capsicum chinense*	Ascentis Express C_30_ (150 mm × 4.6 mm, 2.7 μm)	MeOH	10 min; gradient elution	MS	Qualitative Quantitative	[25]
**Terpenes**	5 triterpenes	*Rosa sericea* (leaf)	HSS C_18_ SB column (100 mm × 3 mm, 1.8 μm)	MeOH with 0.08% TFA	17 min; gradient elution	ELSD	Quantitative	[72]
	8 triterpenoids	Apple pomace extracts	Synergi Polar-RP (250 mm × 4.6 mm, 4 μm)	MeOH	isocratic elution: 3% modifier	ELSD	Qualitative	[73]
	6 sesquiterpenes	*Matricaria chamomilla* (flower), *Chamaemelum nobile* (flower)	ACQUITY UPC^2^ BEH 2-EP column (150 mm × 3 mm, 1.7 μm)	MeOH:IPA (1:1) with 0.5% FA	15 min; gradient elution	PDA and MS	Qualitative	[28]
	Camphor	*Tanacetum parthenium* (seed)	Acquity UPC^2^ BEH-2EP column (100 mm × 3 mm, 1.7 μm)	IPA	10 min; gradient elution	DAD	Quantitative	[74]
	Continentalic acid and kaurenoic acid	*Aralia continentalis* (root) *A. pubescens* (root)	Acquity UPC^2^ Torus 1-AA column (150 mm × 2.1 mm, 1.7 μm)	MeOH with 0.1% FA	isocratic elution: 3% modifier	DAD	Quantitative	[75]
	18 diterpene esters	*Euphorbia semiperfoliata*	Hypercarb column (Carbon, 100 mm × 2.1 mm, 3 μm)	EtOH with 0.1% FA	20 min; gradient elution	DAD and MS	Qualitative	[76]
	5 terpene lactones, 4 ginkgolic acids	*Ginkgo biloba*	ACQUITY UPC^2^ BEH 2-EP column (150 mm × 3 mm, 1.7 μm)	MeOH:IPA (50:50) with 10 mM ammonium acetate	12 min; gradient elution	PDA and MS	Quantitative	[77]
	12 limonoid aglycones	Citrus essential oil	Ascentis C_18_ column (250 mm × 4.6 mm, 5 μm)	MeOH	20 min; gradient elution	MS	Qualitative Quantitative	[78]
	2 triterpenoid acids	*Chaenomelis Fructus* (fruit)	Shim-pack UC-X Diol Column (150 mm × 4.6 mm, 3 μm)	MeOH	20 min; gradient elution	UV	Quantitative	[79]
**Saponins**	Triterpenoid saponins: 9 kudinosides, 6 stauntosides, and 11 ginsenosides	*Ilex latifolia* (leaf), *Panax quinquefolius* (root), *P. ginseng* (root)	ZORBAX RX-SIL column (150 mm × 4.6 mm, 5 μm)	B1: MeOH with 0.05% FA and 10% H_2_O; B2: MeOH with 0.05% FA and 5% H_2_O	Kudinosides: 15 min; Stauntosides: 20 min; Ginsenosides: 17 min; gradient elution	DAD and MS	Qualitative	[32]
	10 furostanol saponins	*Dioscorea zingiberensis* (rhizome)	Diol column (150 mm × 3 mm, 1.7 μm)	MeOH with 0.2% NH_3_·H_2_O and 3% H_2_O	15 min; gradient elution	MS	Qualitative	[33]
	7 ginsenosides, 6 nucleosides, 4 nucleobases	Ginseng, Korean ginseng, American ginseng	ZORBAX RX-SIL column (150 mm × 4.6 mm, 5 μm)	MeOH with 5 mM ammonium acetate	20 min; gradient elution	DAD and MS	Qualitative	[80]
	6 ginsenosides	*Panax quinquefolius* (root)	Cyanopropyl packed column (250 mm × 4.6 mm, 5 μm)	MeOH with 0.05% TFA	18 min; gradient elution	ELSD	Qualitative	[81]
	Paeoniflorin, albiflorin, benzoyl paeoniflorin, oxypaeoniflorin, gallic acid, and benzoic acid	Raw, wine-baked, and vinegar-baked *Paeonia lactiflora* (root)	Acquity UPC^2^ HSS C_18_ SB column (100 mm × 3.0 mm, 1.8 μm)	MeOH:ACN (70:30) with 0.1% phosphoric acid	12 min; gradient elution	PDA	Quantitative	[82]
	Six 25(*R*/*S*)-spirostanol saponin diastereomers	*Trigonella foenum-graecum* (seed)	CHIRALPAK IC column (250 mm × 4.6 mm, 5 μm) couple CHIRALPAK IC column (150 mm × 4.6 mm, 5 μm)	MeOH	isocratic elution: 33% B	ELSD	Qualitative	[83]
**Cannabinoids**	9 cannabinoids	*Cannabis sativa* (flowering bud)	ACQUITY UPC^2^ BEH 2-EP column (150 mm × 3 mm, 1.7 μm)	IPA:ACN (80:20) with 1% H_2_O	10 min; gradient elution	PDA and MS	Quantitative	[84]
	11 cannabinoids	*Cannabis sativa* (flowering bud, hashish, and leaf)	ACQUITY UPC^2^ BEH 2-EP column (150 mm × 3 mm, 1.7 μm)	IPA:ACN (80:20) with 1% H_2_O	10 min; gradient elution	PDA and MS	Quantitative (9); Qualitative (2)	[85]
	7 cannabinoids	*Cannabis sativa*	UHPC-(*S*, *S*)-Whelk-O1 column; UHPC-(*R*, *R*)-Whelk-O1 column (100 mm × 4.6 mm, 1.8 μm)	MeOH	isocratic elution: 2% modifier	UVD and CD	Qualitative	[39]
**Flavonoids**	5 flavonoids	*Chrysanthemum morifolium*	ZORBAX RX-SIL column (150 mm × 4.6 mm, 5 μm)	MeOH with 0.1% phosphoric acid	20 min; gradient elution	DAD	Qualitative Quantitative	[61]
	7 flavonoids	*Astragalus membranaceus* (root)	Acquity UPC^2^ CSH fluorophenyl column	MeOH	13 min; gradient elution	PDA	Qualitative Quantitative	[86]
	6 flavonoids	*Citrus reticulata* (pericarp)	Zorbax RX-SIL column (150 mm × 2.1 mm, 5 µm)	MeOH	11 min; gradient elution	DAD	Quantitative	[87]
	6 flavonoid aglycones	/	Poroshell 120 EC-CN (100 mm × 3 mm, 2.7 μm)	MeOH with 20 mM [bmim][BF_4_]	isocratic elution: 8% modifier	DAD	Qualitative	[64]
	6 flavonoids	*Glycyrrhiza uralensis*, *G. glabra*, and *G. inflata* (root and rhizome)	ACQUITY UPC^2^ Torus 2-PIC column (100 mm × 2.1 mm, 1.7 μm)	MeOH with 0.2% FA	8 min; gradient elution	PDA	Quantitative	[88]
	51 flavonoids, 7 prenyl flavonoids	*5 Epimedium* species (leaf)	Acquity UPC^2^ Torus Diol (100 mm × 3 mm, 1.7 μm)	MeOH:ACN (50:50) with 5 mM oxalic acid and 3% H_2_O	MS: 18 min; gradient elution; PDA: 17 min; gradient elution	MS; PDA	Qualitative (51) Quantitative (7)	[63]
	16 isoflavones	*Pueraria lobata, P. thomsonii, P. peduncularis* (root)	Acquity Torus Diol column (100 mm × 3 mm, 1.7 μm)	MeOH with 1 mM oxalic acid	12 min; gradient elution	PDA and MS	Quantitative	[89]
	9 isoflavones	Dietary supplements containing *Glycine max* (bean), *Trifolium pratense* (blossom), and *Pueraria lobata* (root)	Acquity UPC^2^ BEH column (100 mm × 3 mm, 1.7 μm)	MeOH with 0.05% phosphoric acid	15 min; gradient elution	PDA	Qualitative	[62]
	17 flavonoids and polyphenols	Sweet potato leaf	Acquity UPC^2^ BEH 2-EP column (100 mm × 3 mm, 1.7 μm)	MeOH with 0.05% FA	10 min; gradient elution	MS	Quantitative	[90]
	3 flavonoids, 2 phenolic acids	*Medicago sativa*	ACQUITY UPC^2^ BEH column (100 mm × 3 mm, 1.7 µm)	MeOH	10 min; gradient elution	PDA	Qualitative	[91]
	Xanthohumol	Hop extracts	Waters Symmetry C18 column (250 mm × 4.6 mm, 5 μm); Agilent Zorbax SB C18 column (150 mm × 4.6 mm, 3.5 μm)	EtOH	5 min; gradient elution	DAD	Qualitative	[92]
**Phenolics**	6 phenolics	Liquidambaris (resin)	Acquity UPC^2^ BEH 2-EP Column (100 mm × 3 mm, 1.7 µm)	MeOH with 0.1% phosphoric acid	5 min; gradient elution	PDA	Quantitative	[93]
	9 phenolic compounds	*Allium sativum*	Shim-pack UC-X Diol column (150 mm × 4.6 mm, 3 μm)	MeOH containing 0.1 mM oxalic acid and 1 mM ammonium formate	8 min; gradient elution	MS	Qualitative Quantitative	[55]
	12 phenolic acids	Extra-virgin olive oil	Platisil CN column (250 mm × 4.6 mm, 5 μm)	MeOH containing 7% water and 0.5% FA	30 min; gradient elution	DAD and MS	Quantitative	[94]
	7 phenolic acids	*Lonicera japonica* (flower bud)	ACQUITY UPC^2^ BEH (100 mm × 3 mm, 1.7 μm)	MeOH:ACN (70:30) with 1% TFA	20 min; gradient elution	PDA	Quantitative	[95]
	4 polyphenols	Bee pollen sample	DCpak PBT (250 mm × 4.6 mm, 5 μm)	MeOH with 0.1% TFA	24 min; gradient elution	PDA	Qualitative Quantitative	[96]
	4 lignans	*Schisandra chinensis* (fruit)	Shim-pack UC-X SIL column (150 mm × 2 mm, 3 μm)	MeOH	7.5 min; gradient elution	PDA	Quantitative	[97]
	9 lignans	*Schisandra chinensis* (fruit)	Viridis HSS C_18_ SB column (100 mm × 3 mm, 1.8 μm)	MeOH	12 min; gradient elution	PDA	Qualitative	[98]
	8 vitamin E isomers	*Moringa oleifera* (leaf)	Acquity UPC^2^ BEH 2-EP (100 mm × 3 mm, 1.7 μm)	MeOH:IPA (1:1, *v*/*v*)	6.2 min; gradient elution	DAD	Quantitative	[99]
**Alkaloids**	2 oxindole alkaloids, 6 indole alkaloids	*Mitragyna speciosa* (leaf)	Agilent Rx-Sil column (50 mm × 2.1 mm, 1.8 μm)	MeOH with 10 mM ammonium acetate	10 min; gradient elution	DAD	Qualitative	[17]
	71 sesquiterpene pyridine alkaloids	*Tripterygium wilfordii* (root bark)	ACQUITY UPC^2^ BEH 2-EP column (50 mm × 2.1 mm, 1.7 μm)	MeOH	10 min; gradient elution	DAD and MS	Qualitative	[31]
	Four 7-epimeric spiro oxindole alkaloids	*Uncaria macrophylla*	Torus 1-AA column (100 mm × 3 mm, 1.7 μm); Torus Diol column (100 mm × 3 mm, 1.7 μm)	Torus 1-AA column: ACN with 0.1% diethylamine; Torus Diol column: ACN with 0.1% ammonium hydroxide	Torus 1-AA column: isocratic elution: 22% modifier; Torus Diol column: isocratic elution: 21% modifier	PDA	Qualitative	[66]
	6 cinchona alkaloids	*Cinchona* (bark)	Acquity UPC^2^ Torus DEA column (100 mm × 3 mm, 1.7 μm)	MeOH:ACN (90:10) with 0.8% diethylamine	isocratic elution: 10 min, 2.3% modifier	PDA	Quantitative	[100]
	8 isoquinoline alkaloids	*Mahonia bealei* (stem, root, leaf, and seed)	Inspire PFP column (250 mm × 4.6 mm, 5 μm)	MeOH with 0.2% ammonia solution and 8% H_2_O	isocratic elution: 20 min 25% modifier	DAD	Qualitative Quantitative	[67]
	10 isoquinoline alkaloids	*Sinomenium acutum* (stem rattan), *Corydalis yanhusuo* (rhizome), *Coptis chinensis*, *C*. *deltoidea*, *C*. *teeta* (rhizome), *Mahonia bealei*, *M*. *fortune* (stem), *Phellodendron chinense* (bark), *Stephania tetrandra* (root)	Zorbax RX-SIL column (150 mm × 2.1 mm, 5 µm)	MeOH with 0.25% ChCl-Gly-0.5% FA-2% H_2_O	26 min; gradient elution	DAD	Qualitative Quantitative	[68]
	5 aconitum alkaloids	*Aconitum pendulum* (root)	Acquity UPC^2^ BEH 2-EP (150 mm × 2.1 mm, 1.7 μm)	10 mM ammonium acetate in MeOH	4 min; gradient elution	PDA and MS	Quantitative	[101]
**Miscellaneous**	9 natural aromatic acids	Grape and fruit wines	BEH-2EP column (150 mm × 3 mm, 1.7 μm)	MeOH with 0.1% TFA	3.5 min; gradient elution	DAD	Quantitative	[102]
	5 coumarins	*Ammi visnaga* (fruit)	Acquity UPC^2^ HSS C_18_ SB (100 mm × 3 mm, 1.8 μm)	MeOH:ACN (1:1) with 0.1% diethylamine	7.5 min; gradient elution	PDA	Quantitative	[103]
	8 coumarins	*Angelica dahurica* (root)	Acquity UPC^2^ CSH Fluoro-Phenyl (100 mm × 3 mm, 1.7 μm)	MeOH with 0.1% diethylamine	8 min; gradient elution	PDA	Qualitative Quantitative	[104]
	Decursinol angelate and decursin	*Angelica gigas* (root)	Acquity UPC^2^ CSH Fluoro-Phenyl (150 mm × 2.1 mm, 1.7 μm)	EtOH	isocratic elution: 5% modifier	PDA	Quantitative	[105]
	10 annonaceous acetogenins	*Annona muricata* (fruit)	Acquity UPC^2^ BEH 2-EP Column (100 mm × 2.1 mm, 1.7 μm)	EtOH	16 min; gradient elution	MS	Qualitative	[106]
	Curcumin, demethoxycurcumin, and bisdemethoxycurcumin	*Curcuma longa* (rhizome)	ACQUITY UPC^2^ BEH column (100 mm × 3 mm, 1.7 μm)	MeOH with 10 mM oxalic acid	6 min; gradient elution	PDA	Qualitative	[107]
	5 anthraquinones	*Rheum palmatum* and *R. officinal*e (root)	Acquity UPC^2^ HSS C_18_ SB (100 mm × 3 mm, 1.8 μm)	MeOH with 0.05% diethylamine	10 min; gradient elution	PDA	Quantitative	[108]
	6 kavalactones	*Piper methysticum* (root)	Acquity UPC^2^ BEH-2EP column (100 mm × 3 mm, 1.7 μm)	MeOH with 0.6% diethylamine	5.5 min; gradient elution	PDA	Quantitative	[109]
	4 aromatic constituents	Cured vanilla beans	Shimpack UC-X RP column (150 mm × 4.6 mm, 3 μm)	MeOH	17 min; gradient elution	DAD	Quantitative	[110]
	9 phenylamides	*Piper kadsura*	Torus DIOL (100 mm × 3 mm, 1.7 μm)	MeOH	15 min; gradient elution	MS	Qualitative	[111]
	*R*/*S*-goitrin	*Isatis indigotica* (root), *Baphicacanthus cusia* (root), Ban Lan Gen powder formulations	(*S*, *S*)-Whelk-O1 column (250 mm × 4.6 mm, 10 μm)	MeOH	6 min; gradient elution	PDA and MS	Qualitative Quantitative	[38]
	Seven 25*R*/*S*-ergostanes	*Antrodia camphorata* (fruiting body)	Chiralcel OJ-H column (250 mm × 4.6 mm, 5 μm); Princeton 2-ethylpyridine column (250 mm × 4.6 mm, 3 μm)	MeOH	Chiralcel OJ-H column: 15 min; gradient elution; Princeton 2-ethylpyridine column: 20 min; gradient elution	DAD	Qualitative	[112]
	11 common peaks	*Mahonia bealei*	Platisil NH_2_ (250 mm × 4.6 mm, 5 µm)	MeOH with 0.4% diethylamine and 8% H_2_O	25 min; gradient elution	DAD	Qualitative	[113]
	34 common peaks	*Hedysarum polybotrys* (root)	HSS SB C_18_ column (150 mm × 2.1 mm, 1.7 μm)	MeOH with 0.2% FA	23 min; gradient elution	DAD	Qualitative	[114]

“i.d.”: internal diameter; “dp”: diameter of particles; “Ref.”: reference; “–”: not mentioned; “DAD”: diode array detector; “PDA”: photo-diode array detector; “MS”: mass spectrometry; “ELSD”: evaporative light scattering detector.

**Table 2:** Application of SFC in series with other chromatography techniques in the analysis of natural products.

No.	Compounds	Species	Type	^1^D Column (length × i.d., dp)	^2^D Column (length × i.d., dp)	Detector	Ref.
1	amide alkaloids	*Piper longum*	Offline SFC/RPLC	XAmide column (150 mm × 4.6 mm, 5 μm) CO_2_/MeOH	Acquity HSS T3 (100 mm × 2.1 mm, 1.8 μm) H_2_O/ACN	UV	[117]
2	lignans	*Fructus Arctii*	Offline SFC/RPLC	XAmide column (250 mm × 20 mm, 10 μm) CO_2_/MeOH	Unitary C18 column (250 mm × 10 mm, 5 μm) H_2_O/ACN	UV-Vis	[118]
3	triterpene saponins	*Panax notoginseng* (stem)	Offline SFC/RPLC	Atlantis HILIC column (150 mm × 4.6 mm, 5 μm) CO_2_/MeOH	Agilent Poroshell EC-C18 (50 mm × 3 mm, 2.7 μm) H_2_O/ACN	PDA; UV-MS	[119]
4	carotenoid and chlorophyll	*Capsicum annuum*	Offline SFC/RPLC	Acquity HSS C18 SB column (100 mm × 3 mm, 1.8 μm) CO_2_/EtOH	YMC C30 column (250 mm × 4.6 mm, 3.0 μm) MeOH:MTBE:H_2_O (86:12:2, *v*/*v*/*v*)/MeOH:MTBE:H_2_O, (8:90:2, *v*/*v*/*v*)	ELSD; PDA-MS	[120]
5	carotenoids	*Capsicum annuum*	Online SFC × RPLC	Ascentis ES Cyano (250 mm × 1.0 mm, 5.0 μm) CO_2_/MeOH	Acquity BEH C18 (50 mm × 2.1 mm, 1.7 μm) ACN:H_2_O (8:2 (*v*/*v*))/IPA	PDA-MS	[121]
6	psoralens and coumarins	Lemon oil	Online SFC × RPLC	Four Princeton SFC cyanopropyl silica columns (250 mm × 2 mm, 5 μm) CO_2_/EtOH	Zorbax SB C18 (50 mm × 4.6 mm, 3.5 μm) H_2_O/ACN	DAD	[122]
7	bufadienolides	*Bufo gargarizans or B. melanostrictus* (secretion)	Offline RPLC/SFC	HSS T3 column (250 mm × 4.6 mm, 5 μm) 0.1% FA-water/acetonitrile	ACQUITY UPC^2^ HSS C18 column (100 mm × 3.0 mm, 1.8 μm) CO_2_/MeOH	UV; PDA-MS	[123]
8	/	Blackberry sage fragrant oil	Offline RPLC/SFC	Phenomenex Luna C18 (150 mm × 4.6 mm, 3 μm) CO_2_/ACN	PrincetonSFC Amino column (250 mm × 4.6 mm, 10 μm) H_2_O/ACN	UV	[124]
9	secondary metabolites	*Ganoderma lucidum* (fruiting body)	Online NPLC × SFC	Hypersil-CN column (200 mm × 4.6 mm, 5 μm) Hexane/isopropanol	Merck Chromolith Flash C18 (50 mm × 4.6 mm) CO_2_	UV	[125]

“i.d.”: internal diameter; “dp”: diameter of particles; “Ref.”: reference; “–”: not mentioned. “DAD”: diode array detector; “PDA”: photo-diode array detector; “MS”: mass spectrometry; “ELSD”: evaporative light scattering detector.

## Data Availability

Not applicable.

## Sample Availability

Samples of the compounds are not available from the authors.
